# Safety of flexible endoscopic biopsy of the pharynx and larynx under topical anesthesia

**DOI:** 10.1007/s00405-017-4647-z

**Published:** 2017-06-21

**Authors:** David J. Wellenstein, Joey K. de Witt, Henrieke W. Schutte, Jimmie Honings, Frank J. A. van den Hoogen, Henri A. M. Marres, Robert P. Takes, Guido B. van den Broek

**Affiliations:** 1 Department of Otorhinolaryngology and Head and Neck Surgery, Radboud University Medical Center, Philips van Leydenlaan 15, 6500 HB Nijmegen, The Netherlands; 2 Department of Surgery, The Netherlands Cancer Institute, Antoni van Leeuwenhoek Hospital, Amsterdam, The Netherlands

**Keywords:** Flexible endoscopic biopsy, Laryngeal biopsy, Office-based biopsy, In-office biopsy, Office-based procedures, Local anesthesia

## Abstract

Recent advancements in transnasal endoscopy enable a shift in diagnostic workup of lesions in the pharynx and larynx, from an examination with biopsy under general anesthesia to an office-based examination with flexible endoscopic biopsy under topical anesthesia. Procedural complications were evaluated to assess the safety of office-based flexible endoscopic biopsy in patients with benign and malignant laryngopharyngeal lesions. Patients who underwent flexible endoscopic biopsy from 2012 to 2016 were evaluated retrospectively. Complications were classified using the Clavien–Dindo classification of surgical complications. A total of 201 flexible endoscopic biopsies were performed in 187 patients. Two Clavien–Dindo grade I (laryngospasm and anterior epistaxis), one grade II (laryngeal bleeding), and one grade IIIb (laryngeal edema) complication were observed. The first complication was self-limiting and the other three required an intervention. All patients fully recovered without sequelae. Flexible endoscopic biopsy appears to be a safe office-based procedure for the diagnosis of benign and malignant laryngopharyngeal lesions.

## Introduction

Head and neck cancer is the fifth most common cancer in the world [1]. The estimated incidence of nasopharyngeal, oropharyngeal, hypopharyngeal, and laryngeal (i.e., laryngopharyngeal) cancers worldwide was approximately 385,000 new cases in 2012 with over 230,000 estimated deaths [1]. In The Netherlands, approximately 1565 new cases of laryngopharyngeal cancer were diagnosed in 2015 [2].

The early diagnosis is crucial for improving the treatment results of laryngopharyngeal cancer [3]. It increases survival and the chance of preserving laryngeal function [4]. Diagnostic assessment aims at histological diagnosis, mapping, and staging of the tumor [3]. The initial work-up starts with a history, physical examination, and imaging [5]. However, a biopsy is essential for the histological diagnosis of laryngopharyngeal cancer [3]. Traditionally, the biopsy of laryngopharyngeal lesions is performed under general anesthesia. Technological advancements in the types of transnasal endoscopes, instrument miniaturization, and topical anesthetic techniques have led to a shift in laryngeal management from the operation room to an office-based setting [6–8]. Since the introduction of fiberoptic laryngoscopy in the 1970s, lighting and imaging techniques have improved substantially [9]. During the last decade, fiberoptic endoscopy has gradually been replaced by distal chip endoscopy [10, 11]. In the latter, information from a chip in the distal tip of the endoscope is send to a video processor, which creates a digital image and enables high-resolution imaging. Furthermore, endoscopes can be equipped with a built in working channel for passage of a flexible biopsy forceps or a laser fiber [6, 8, 10]. This enables clinicians to perform surgical procedures under topical anesthesia in an office-based setting, such as laser surgery or flexible endoscopic biopsy (FEB).

Office-based laryngeal FEB is reported to be safe [6, 12–14], feasible [6], cost-effective [12, 15], and easy to perform [13, 16]. The advantages are an awake patient who is sitting in an upright position and able to control laryngeal function during the procedure, which can result in adequate visualization of the designated lesion [10]. Furthermore, there is avoidance of general anesthesia with possible health benefits [14, 17]. Its costs are relatively low compared with examination and biopsy under general anesthesia [12, 15]. Most importantly, FEB can often be performed during the initial outpatient visit or follow-up visit, which results in reduced diagnostic delay [7, 18]. An additional advantage of digital endoscopic techniques is the possibility of recording images, enabling more detailed reporting in the patient’s electronic file and comparison of images during follow-up. The disadvantages of laryngeal FEB are the need for a cooperative patient (e.g., able to sit still, minimal gag reflex) [8] and the inability to perform deep biopsies of submucosal tumors [12].

Due to the relative novelty of this office-based procedure, few studies are available and complication rates have been assessed in small sample sizes [6, 7, 12–14, 19]. Therefore, the aim of this study was to investigate the complications of transnasal FEB under topical anesthesia in patients with benign and malignant laryngopharyngeal lesions. This study provides a more detailed insight into the safety of the procedure. To assess safety, complications were evaluated using the Clavien–Dindo classification, which is an objective scoring system for classifying complications and is also reproducible [20]. To our knowledge, the investigated study population is the largest in the literature.

## Materials and methods

### Patient selection

This retrospective cohort study was conducted from April 2012 to April 2016 at the Department of Otorhinolaryngology and Head and Neck Surgery of the Radboud University Medical Center in Nijmegen, The Netherlands. Eligible study participants were patients aged 18 years or older who underwent transnasal FEB under topical anesthesia for benign or malignant laryngopharyngeal lesions. Anticoagulant (i.e., thrombocyte aggregation inhibitors, adenosine diphosphate receptor inhibitors, and vitamin K antagonists) use was not considered a contraindication. Submucosal lesions or lesions with undefined tumor margins visualized during diagnostic flexible laryngoscopy were excluded. These patients were excluded, because the FEB procedure was performed not only to obtain a histological diagnosis, but also to provide information on tumor size and stage. Patients who underwent multiple biopsies for recurrent lesions or for two separate suspected laryngopharyngeal lesions were included twice.

### Diagnostic work-up

**Fig. 1:**
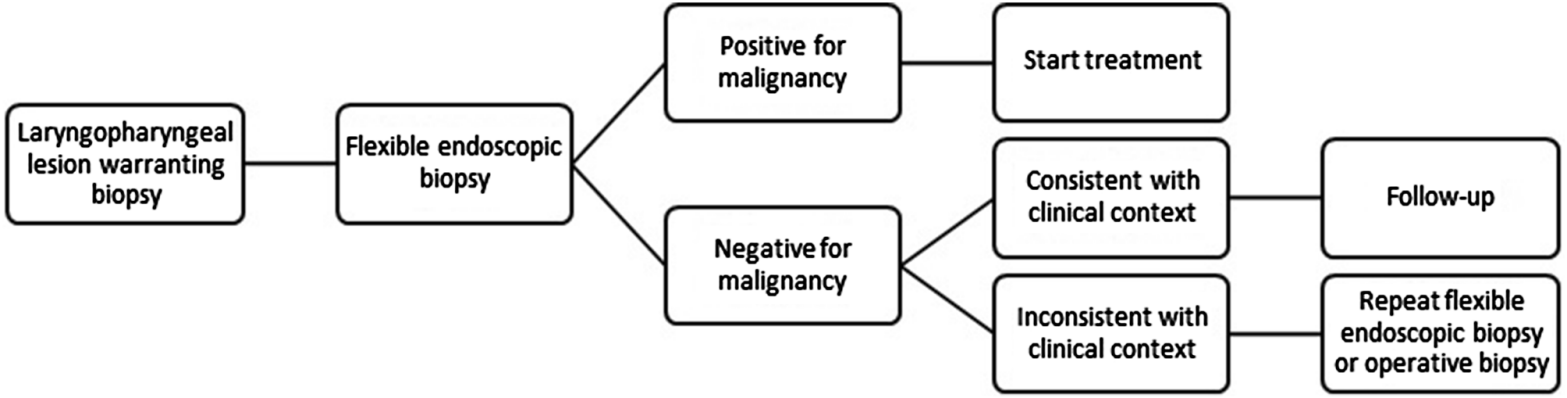
Flowchart of flexible endoscopic biopsy

In this study, FEB was the first choice for diagnostic work-up of benign and malignant laryngopharyngeal lesions, as shown in Fig. 1. Patients visiting the outpatient clinic of our department usually receive FEB the same day. As all other diagnostic (imaging) procedures are performed within 2 days and the results of histological and cytological examination are available within 1 day, each case can be discussed in the multidisciplinary head and neck oncology tumor board meeting after 2 days. The histology result of laryngeal FEB was positive for malignancy (i.e., malignant result), negative for malignancy (i.e., nonmalignant result), or non-diagnostic. A non-diagnostic result could occur when it was impossible to obtain a biopsy specimen or when no histological diagnosis could be determined in case of superficially obtained tissue [15]. In the case of an established malignancy, the treatment could be determined by the multidisciplinary tumor board immediately. In contrast, nonmalignant results of FEB were interpreted with caution. In case of an inconsistency with the clinical findings (i.e., appearance suspicious for malignancy), FEB was repeated or an examination with biopsy under general anesthesia was performed. If a nonmalignant result of FEB that was consistent with the clinical findings was obtained, the patient proceeded to follow-up according to protocol.

### Biopsy technique

After acquiring informed consent from the patients, FEB was performed in an examination room at the outpatient clinic department. Prior to biopsy, topical anesthesia was administered using two cotton pledgets and soaked in 10% lidocaine spray and 0.1% xylometazoline solution, which were placed in each side of the nasal cavity for a minimum of 10 min. The pharynx and larynx were sprayed with 10% lidocaine spray transorally, immediately after nasal anesthesia and just prior to the procedure. The number of sprays varied between 8 and 14. In case of a laryngeal biopsy, additional anesthesia was performed by intratracheal injection of 1.0 ml of 10% lidocaine through the cricothyroid membrane and topical administration on the vocal cords.

Biopsies were obtained using a flexible video endoscope with a 2.0 mm diameter working channel (VNL-1570STK, Pentax Medical, Japan) and a single-use 1.8 mm diameter flexible biopsy forceps (Radial Jaw™ 4, Boston Scientific, Costa Rica). Images were displayed and recorded using a video processor (EPK-i5000, Pentax Medical, Japan). During biopsy, the flexible video endoscope was inserted transnasally in patients in a sitting position. After visualization of the laryngopharyngeal lesion, a biopsy forceps was passed through the working channel. The lesion was approached with an open jaw biopsy forceps, pushed deep into the lesion, and closed. The biopsy forceps was pulled back swiftly, while feeling for resistance when removing the tissue from the suspected lesion, which indicates a deeply taken biopsy. If possible, three deep biopsies were taken from each suspected lesion. All biopsies were performed by two physicians, either head and neck surgeons or senior residents under direct supervision.

### Data collection and analysis

Data were collected on patient characteristics (i.e., sex, age, anticoagulation use, biopsy site, and definitive histological diagnosis) and complications. A complication was defined as any deviation from the normal course after the performance of a FEB procedure [20]. To minimize subjectivity in the reporting of complications, terms such as ‘minor’, ‘major’, ‘mild’, or ‘severe’ were avoided. Instead, the Clavien–Dindo classification of surgical complications was used [20]. Statistical analysis was performed using IBM Statistical Package for Social Sciences Statistics 22 (IBM Corp. Released 2013. IBM SPSS Statistics for Windows, Version 22.0. Armonk, NY: IBM Corp).

## Results

Two hundred and one transnasal FEB procedures in 187 patients were included with 561 obtained biopsies, resulting in an average of 2.8 biopsies per procedure. There were 14 double inclusions, of which 13 were due to suspected recurrent lesions and one patient had two separate tumors that required FEB.

Patient characteristics are shown in Table 1. The study population consisted mostly of men (78.6%) of older age (mean 66.8 years). Almost one-third of patients used anticoagulants (29.9%). The most common biopsy site was the larynx with 138 biopsies (68.7%). Definitive histology results were squamous cell carcinoma in over half of the cases (57.2%) and severe dysplasia or carcinoma in situ in over one-fifth of the cases (21.4%). One histology result showed a lymphoma. In 5 out of 201 procedures, it was impossible to obtain a biopsy specimen. In one patient, the flexible endoscope could not be passed through the nose due to the small nasal anatomy of the patient; in two patients, a complication occurred during biopsy and the procedure, therefore, had to be aborted (i.e., laryngeal bleeding and supraglottic edema); in two cases, the patient could not tolerate the procedure (e.g., excessive coughing or pharyngeal reflex). In 1 out of 201 FEB procedures, histological classification could not be determined by the pathologist because of superficial biopsy specimens.

**Table 1:** Patient characteristics of laryngopharyngeal flexible endoscopic biopsy

Characteristics	Flexible endoscopic biopsy	%
Study population	201	100
Sex (number males)	158	78.6
Age (range)	66.8 (43–92)	
Anticoagulant use (no)	60	29.9
Site biopsy		
Nasopharynx (no)	5	2.5
Oropharynx (no)	36	17.9
Hypopharynx (no)	19	9.5
Larynx (no)	138	68.7
Neopharynx (no)	3	1.5
Definitive histology		
Benign (no)	5	2.5
Hyperplasia	24	11.9
Mild/moderate dysplasia	7	3.5
Severe dysplasia/carcinoma in situ	43	21.4
Squamous cell carcinoma (no)	115	57.2
Lymphoma (no)	1	0.5
No histology result (no)	6	3.0

In 201 FEB procedures that were performed in this study, four patients had complications. Complications were laryngospasm, anterior epistaxis, laryngeal bleeding, and supraglottic edema; using the Clavien–Dindo classification system, these complications could be defined as two grade I (laryngospasm and anterior epistaxis), one grade II (laryngeal bleeding), and one grade IIIb (supraglottic edema) complication, respectively. The laryngospasm was self-limiting, while the other complications required an intervention. All complications resolved without sequelae.

### Case 1: Laryngospasm

A 61-year-old man was referred to us with a neck metastasis from an unknown primary tumor. During diagnostic flexible laryngoscopy, a small ulcerative lesion was observed on the laryngeal side of the epiglottis. The patient underwent a FEB, and after the first biopsy, laryngospasm with stridor occurred. The patient was attended in the outpatient clinic and the symptoms were self-limiting. No further biopsies were performed. The patient underwent an examination under general anesthesia that did not reveal a malignancy. The tumor was defined as an unknown primary and the patient underwent treatment according to protocol.

### Case 2: Anterior epistaxis

A 63-year-old woman underwent a FEB for a suspicious lesion in the vallecula with extension up to the epiglottis. After the first biopsy, the patient developed anterior epistaxis. Intranasal cotton pledgets soaked in 0.1% xylometazoline were placed in the right nostril. The anterior epistaxis stopped and three more biopsies were performed.

### Case 3: Laryngeal bleeding

A 73-year-old woman was diagnosed with an intubation granuloma, which she developed after surgery 2 months earlier. During examination under general anesthesia, adequate visualization of the intubation granuloma was not possible because of the small mandibular space and limited retroflexion of the neck. Therefore, a FEB was scheduled. After injecting topical anesthesia through the cricothyroid membrane of the glottic region, hemoptysis occurred. There were no symptoms of dyspnea or stridor. Nebulized adrenalin and xylometazoline were ineffective, but the laryngeal bleeding stopped after subcutaneous injection of 0.5 mg of adrenalin around the cricothyroid membrane. The patient had an overnight observation in the hospital. On follow-up 4 weeks later, spontaneous regression of the intubation granuloma was observed during flexible laryngoscopy.

### Case 4: Subglottic edema

A 83-year-old man with a medical history of chronic obstructive pulmonary disease and Alzheimer’s disease was referred by the general practitioner to our hospital because of hoarseness and increasing dyspnea. Treatment with inhalation corticosteroids was started because of suspicion of an exacerbation of the chronic obstructive pulmonary disease. At flexible laryngoscopy, a large bilateral glottic tumor was observed. After taking the first biopsy, the patient experienced a strong pharyngeal reflex and started coughing vigorously. Additional topical anesthesia was administered and the procedure was temporarily paused. It was not possible to proceed with the FEB because of coughing, and the patient was dismissed from the hospital without additional complaints.

The same evening, the patient was admitted to the emergency department with increasing dyspnea and laryngeal edema was seen during flexible laryngoscopy. He was repeatedly nebulized with 30 ml of salbutamol/ipratropium at 0.1/1.0 mg and administered 0.5 mg adrenalin intravenously. The effect was insufficient, and the patient underwent a tracheotomy in the operation room with biopsies the next day, which histologically showed squamous cell carcinoma of the vocal cords.

## Discussion

In the last decade, technological advancements have caused a shift in the evaluation of most laryngopharyngeal lesions from the operating room to an office-based setting [6, 8]. However, before a diagnostic method is implemented in clinical practice, several factors need to be considered [14]. First, the safety of office-based FEB for laryngopharyngeal lesions has to be determined. The current study showed that four complications occurred out of 201 FEB procedures, and each complication did not occur twice. Thus, the incidence was 0.5% for each complication. A few studies reported on the safety of FEB for laryngopharyngeal lesions, showing low complication rates [6, 7, 12–14]. Cohen et al. reported three complications in 112 patients, including “a post-procedure aspiration in one patient (without serious consequences) and a self-limited epistaxis in two patients” [13]. Further description, for example, on the intervention or outcome of these complications, was not provided by the authors. Lippert et al. assessed the safety of FEB in 24 transoral and 76 transnasal procedures [14]. No complications were reported and no association was observed with procedure tolerance or biopsy approach. Three other articles, with patient numbers varying from 12 to 76 patients, reported no complications during FEB [6, 7, 12].

To our knowledge, this is currently the largest study cohort for FEB reported in the literature. Furthermore, complications were systematically identified and classified according to a validated protocol. The results of the current study are in line with those of previous studies regarding the complications of FEB for laryngopharyngeal lesions, and showed four complications in a total of 201 procedures. Grade I and II complications, anterior epistaxis and laryngeal bleeding, demanded intervention as well as the grade IIIb complication of supraglottic edema. The grade I complication of laryngospasm was self-limiting. It is important to note that all complications recovered without sequelae. The case of supraglottic edema resulted in a tracheostomy, which was reversed approximately 2 weeks later.

Contraindications are an important consideration in any surgical procedure. In this study, a patient with a compromised airway was considered an absolute contraindication, which is in agreement with the reported literature [7]. In retrospect, the fourth case may not have had a compromised airway during biopsy, but he did have a compromised airway, which required a tracheotomy, when he was admitted to the emergency department. In some studies, anticoagulant use was considered a relative contraindication; patients were advised to stop anticoagulation use, defer the procedure, or they were excluded from further analysis [6, 7, 14, 15]. In the current study, anticoagulant use was not considered a contraindication and it was reported in 29.9% of the patients who underwent FEB. One of four patients (25%) with a complication in this cohort used anticoagulants but did not develop bleeding. Although care should be taken in patients who use anticoagulants, we believe that this should not be a factor that prevents a FEB under topical anesthesia.

A limitation of this study was the retrospective study design. Furthermore, there was a possibility of selection bias, and some complications may have developed after the outpatient clinic visit, which might not be included in this study. In addition, the physician judged whether a patient could cooperate in FEB under topical anesthesia. This is a subjective measure and it, therefore, might differ between physicians. It is important to note that this would occur less often with more experience gained with the number of laryngeal FEB procedures performed and local anesthetic techniques.

## Conclusion

In this study, FEB for laryngopharyngeal lesions showed low complication rates and we thus conclude that the performance of FEB under topical anesthesia is safe for the histological diagnosis of benign and malignant laryngopharyngeal lesions. These results can contribute to a wider understanding of the possible complications that can occur during FEB and help guide physicians when FEB is first performed in the outpatient clinic.
